# Supplementary material for: Hierarchical self-assembly of 3D lattices from polydisperse anisometric colloids
Source: Nat Commun. 2019 Apr 18;10:1815. doi: 10.1038/s41467-019-09787-6 (PMC6472373; doi:10.1038/s41467-019-09787-6)
Supplement: Supplementary file 1 — Supplementary Information [file 41467_2019_9787_MOESM1_ESM.pdf]

Supplementary Information

Hierarchical Self-Assembly of 3D Lattices from Polydisperse Anisometric Colloids

Luo *et al.*

Supplementary Notes

Supplementary Note 1. Estimation of the fluctuation height of anisometric plates

The fluctuation height h, the height a colloid in a solvent can thermally fluctuate up to against gravity, is defined following a literature convention[1]: $(\rho_{\mathrm{Ag}} - \rho_{\mathrm{water}})Vgh = k_{\mathrm{B}}T$, where ρ_{Ag} and ρ_{water} are the densities of silver (10.49 g cm^{-3}) and water (1.0 g cm^{-3}) respectively, V is the volume of a silver plate, g is the standard gravity, 9.8 m s^{-2}, k_{B} is Boltzmann constant (1.38 × 10^{-23} J K^{-1}) and T is the temperature (298 K). Take the truncated plates (Figs. 1–2) as an example. They have an average basal plane area (S) of 3.27 μm^2 and an average thickness (t_{plate}) of 35 nm, leading to a volume V of 1.14 × 10^{-1} μm^3. For this system, we have h =388 nm, which is as much as 11 times the plate thickness. In Supplementary Figure 2a, we calculated the relative probabilities of plate at different heights (H) based on the Boltzmann distribution ($p_{\mathrm{rel}}(H) \approx e^{-((\rho_{\mathrm{Ag}}-\rho_{\mathrm{water}})VgH)/k_{\mathrm{B}}T}$), which shows the plates can go up to micrometers away from the substrate. In Supplementary Figure 2b which shows the fluctuation height as a function of plate thickness, we varied the plate thickness t_{plate} (in units of nm), while keeping the basal plane area constant (3.27 μm^2). The fluctuation height decreases with increasing plate thickness, suggesting the importance of nanoscale thickness to allow free motions of particles in the vertical direction.

Supplementary Note 2. The deprotonation percentage of carboxylate–thiols

The percentage of deprotonated carboxylate–thiols on the plate surface determines the plate surface charge density. We followed the Henderson–Hasselbalch equation: $\mathrm{pH} = \mathrm{p}K_{\mathrm{a}} + \log(\frac{[\mathrm{A}^-]}{[\mathrm{HA}]})$, where $[\mathrm{A}^-]$ and [HA] are the molar concentrations of –COO$^-$ and –COOH groups. The pK_{a} of the carboxylate–thiols is between 3.5 and 3.7 (according to the manufacturer), and we used 3.5 in our calculation. Based on the equation, over 99% of carboxylate–thiols are deprotonated at a pH higher than 5.5.

Under the experimental condition, the amount of CO_2 dissolved in water under atmosphere pressure can be calculated to evaluate its effects on pH and ionic strength of the solution. Based on Henry's Law[2], the amount of CO_2 dissolved in water is [CO_2(aq)] =$H^{cp}_{\mathrm{CO_2}} P_{\mathrm{CO_2}}$. Here $H^{cp}_{\mathrm{CO_2}}$ is Henry solubility constant for CO_2 in water and $P_{\mathrm{CO_2}}$ is the equilibrium partial pressure of CO_2 in air. Plugging in $H^{cp}_{\mathrm{CO_2}}$ =3.4 × 10^{-2} mol L^{-1} atm^{-1}, and $P_{\mathrm{CO_2}}$=0.000355 atm, we get the concentration of dissolved CO_2, c_0 =1.2 × 10^{-5} mol L^{-1}. The dissolved CO_2 will then react with H_2O: $CO_2(aq) + H_2O \rightleftharpoons H^+ + HCO_3^-$. The (apparent) first dissociation constant follows $K_{\mathrm{a1}} = \frac{[\mathrm{H}^+][\mathrm{HCO_3}^-]}{[\mathrm{CO_2}(aq)]}$ =4.45 × 10^{-7} mol L^{-1} and the second dissociation constant follows K_{a2} =4.69 × 10^{-11} mol L^{-1}. Since $\sqrt{c_0 K_{\mathrm{a1}}} > 40K_{\mathrm{a2}}$ and $c_0 K_{\mathrm{a1}} > 20\, K_{\mathrm{w}}$ (K_{w} is the dissociation constant for water), we can ignore the amount of H^+ from water dissociation and the second dissociation reaction. As a result, we get [H^+] ≈[HCO_3^-] =$\sqrt{(c_0 - [\mathrm{H}^+])K_{\mathrm{a1}}}$ =2.1 × 10^{-6} mol L^{-1}. The solution pH after considering CO_2 dissolving in water is 5.7, which still keeps 99% of the charged ligands fully deprotonated. We expect this pH value would not change the deprotonation of ligands, namely the surface charge density of the plates, and have negligible increase (0.002 mM, 0.4% increase) on the ionic strength of the solution at 0.5 mM NaCl condition.

Supplementary Note 3. Columns standing versus lying on the substrate

We observed experimentally that the columns composed of plates of a relatively large basal plane area (3.27 μm^2), such as those used in Figs. 1–2, tend to favor a "standing" configuration (where the plate basal planes sit parallel to the substrate) and have a lower tendency to orient their basal plane perpendicular to the substrate. "Lying" columns were observed in a small proportion (< 10%). This "standing" configuration is likely due to a combined effect of gravitational force and the van der Waals attraction between plate and glass substrate, which we calculated as below. A silver plate standing perpendicular to the substrate has a

larger center-of-mass height than that lying parallel to the substrate (Δh ~868 nm). As a result, the standing plate experiences a higher gravitational potential of 2.5 $k_{\mathrm{B}}T$. This estimation suggests that the plates are favored to sit parallel to the substrate due to gravity. Regarding van der Waals attraction, the equation between two parallel slabs of different materials[3] is

$$E_{\mathrm{vdW}}(d') = -\frac{H_{1w2}S}{12\pi}\left[\frac{1}{(d')^2} - \frac{1}{\left(d'+t_{\mathrm{plate}}\right)^2} - \frac{1}{\left(d'+t_{\mathrm{glass}}\right)^2} + \frac{1}{\left(d'+t_{\mathrm{plate}}+t_{\mathrm{glass}}\right)^2}\right] \quad (1)$$

Here t_{glass} is the thickness of glass substrate (0.13–0.17 mm), much larger than the thickness of a plate t_{plate} (35 nm), so equation (1) can be simplified to

$$E_{\mathrm{vdW}}(d') = -\frac{H_{1w2}S}{12\pi}\left[\frac{1}{(d')^2} - \frac{1}{\left(d'+t_{\mathrm{plate}}\right)^2}\right] \quad (2)$$

In both equations, d' is the face-to-face separation between the substrate and the plate, S is the plate basal plane area, and H_{1w2} is the Hamaker constant for Ag and silica interacting across water[4] (1.9×10^{-20} J). Based on the calculation, the plate–substrate van der Waals attraction can be about –18 $k_{\mathrm{B}}T$ at a separation of ~100 nm and a few $k_{\mathrm{B}}T$ at ~250 nm separation, to support the columns "standing" on the substrate. On the other hand, when columns are composed of plates with small basal planes (for example, 0.90 μm^2), the lying configuration is preferred at relatively low ionic strengths[5]. Standing configurations are only favored when the ionic strength is high enough to screen the electrostatic repulsion between the columns and the substrate.

Supplementary Note 4. Processing of optical microscopy images

The optical microscopy images in Figs. 1e and 4d were processed as follows[6] to improve the image quality. The raw optical microscopy images were first extracted using ImageJ. Next a small number of sequential frames in the movie were averaged using MATLAB (5 images for Fig. 1e and 5 images for Fig. 4d). The number of sequential frames used for averaging was chosen such that the contrast was improved without loss of spatial resolution due to motion-blurring. The averaged image was further processed to subtract the image background using the built-in function of "Subtract Background" in ImageJ, followed by brightness and contrast adjustment. The rolling ball radius for the background subtraction was set as 1000 pixels. The original, averaged and background subtracted images are shown in Supplementary Figure 5.

Supplementary Note 5. Characterization of the three-dimensional (3D) nature of the assembled lattice

We have the following experimental data to demonstrate the 3D nature of the assembled lattices.

First, the formation of the columns is supported by (i) direct imaging of face-to-face stacking of individual plates into columns as shown in Supplementary Figure 2c and Supplementary Movie 1, and (ii) direct imaging of a rotating column exhibiting different orientations as shown in Fig. 1c and Supplementary Movie 2. Rotating columns exhibit light, rectangular projections while lying on their sides, and dark, polygonal projections while standing vertically on the substrate. The fact that the columns serve as the building blocks of the lattice suggests that the lattice is 3D. Second, the polygonal projections of the standing columns shown in Fig. 1c match with the building blocks in the final hexagonal lattice (Fig. 1e), and we observed the gradual increase of the concentration of standing columns leading to the 3D structure (Fig. 1f). The empty space between each standing column in the 3D structure at intermediate column concentration (Supplementary Movie 4) makes it clear to see the plates stacked upon each other into columns with fast rotations (Supplementary Figure 6c). A similar aspect is shown in Supplementary Figure 10 detailing different plate layers in columns comprising the 3D hexagonal lattice presented in Fig. 2c and Supplementary Movie 6. Lastly, the polygonal projections of the lattice building blocks dynamically change their shapes, which is not possible if the lattice is formed from 2D layer of individual triangular plates.

Plates of the first few layers in the column are identified based on their contrast under optical microscopy in Supplementary Movie 5.

We controllably dried the observed lattice after its formation at 3 mM ionic strength in the optical microscopy chamber, to maximally maintain the structure for scanning electron microscopy (SEM) imaging as we monitored under the optical microscopy (although the plate-to-plate spacing in the same column disappears). In Supplementary Figure 8, the SEM images clearly show that the plates are stacked face-to-face into columns, which further assemble laterally into 3D structures.

Supplementary Note 6. Analysis of the local curvature of column projection contours

Curvature analysis (Figs. 2a–b, 4b) was performed using built-in functions in ImageJ to identify the contours of the column projection, followed by local curvature calculation using MATLAB. Specifically, we used the "Make Binary" function in ImageJ to binarize the optical microscopy image, the "Outline" function in ImageJ to recognize the contour, and the "Wand" function in ImageJ to extract the XY coordinates of this contour, which serve as the input for the curvature calculation in MATLAB[6]. The detailed processes are shown in Supplementary Figure 9a. Sometimes the image threshold was slightly adjusted in order to differentiate a column from nearby single plates, followed by the "Dilate" function to obtain accurate contours. The curvature values ($1/R$) were calculated by fitting a local arc of the contour with circles, where R is the radius of the fitted circle. The fitting was conducted by first smoothing the contour by averaging every successive 5 points, and then finding the circle radius for each point using the nearest 25 points (Fig. 2a–b) or 15 points (Fig. 4b) in the contours depending on the image magnification.

Supplementary Note 7. Analysis of the hexagonal lattice

The analysis workflow of the hexagonal lattice (Fig. 2, Supplementary Movie 3) is shown in Supplementary Figure 11. First the positions of individual columns in each frame were tracked, as the central positions of the fitted circumscribed circles of the column projections. Based on the tracked central positions, we were able to derive a series of parameters. First, we computed the velocities of each column over time increments of 0.7 s (12 frames) as shown in Fig. 2d. Second, we performed a Voronoi cell analysis and calculated the local density ρ_j, which is the inverse of the area of the Voronoi cell enclosing column j. Third, the radial distribution function[7] $g(r)$ was calculated based on the column positions over 32 frames in the movie of the hexagonal lattice (Fig. 2e). We followed the standard method[8] to perform periodic boundary correction of the computed $g(r)$ to account for the limited viewing area. From the $g(r)$ *vs.* r curve, r_c (3.13 μm) located at the first minimum after the first peak of $g(r)$ was obtained to determine the threshold for the nearest-neighbor bond length (Supplementary Figure 11b). In particular, only columns with a center-to-center distance $r < r_c$ are recognized as connected *via* a nearest neighbor bond. A bond network was mapped for each frame in the movie to determine the number of the nearest neighbors (Supplementary Figure 11c). We then computed a six-fold local bond orientation order parameter[9] for each column j following $|\psi_{6j}| = |\frac{1}{Z_j}\sum_{k=1}^{Z_j}\exp(6i\beta_{jk})|$, where Z_j is the number of nearest neighbors of column j, the summation goes over all nearest neighbors, and β_{jk} is the angle between the bond linking column j and its kth neighbor and an arbitrary reference axis. This analysis was performed on each frame in the movie (in total 32 frames). Given the local density ρ_j and structural order parameter $|\psi_{6j}|$, we were able to plot out a two-dimensional (2D) histogram of columns on ($|\psi_{6j}|$, ρ_j) as shown in Fig. 2f.

Supplementary Note 8. Analysis of the honeycomb lattice structures

In a honeycomb lattice domain, we measured the orientations of three long sides of the visible (likely on the first layer) plates in each column. The 2D orientation map of the three long sides of first-layer plates is shown in Fig. 4d, which shows a six-fold symmetry, further confirming the honeycomb lattice structure.

Supplementary Figures

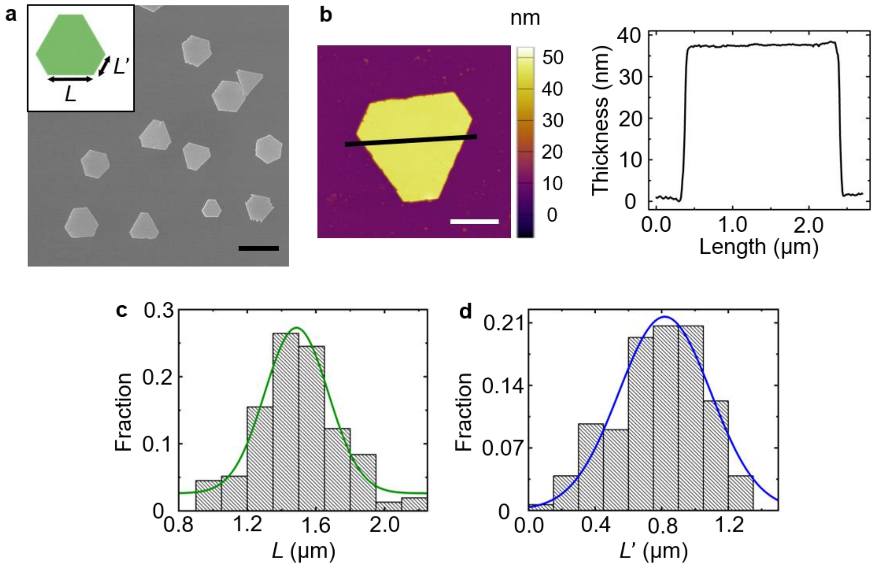

Supplementary Figure 1. Characterization of the anisometric silver plates (long side length *L*: 1.49 ± 0.24 µm, used for the hexagonal lattice, Figs. 1–2). (**a**) A SEM image of the anisometric silver plates. The inset is a schematic of a single silver plate labeled with long side length *L* and short side length *L'*. (**b**) (left) A typical atomic force microscopy (AFM) image and (right) the corresponding thickness profile (across the black line in the AFM image) of a representative silver plate. The color bar represents height. (**c**) and (**d**) The long and short side length distributions of silver plates measured from SEM images using ImageJ. The green and blue curves are the Gaussian fits. Note that our samples contain in general two size populations as discussed in our previous work[5]: large plates that assemble and small plates that do not participate in self-assembly at our typical experimental ionic strength conditions, due to their much smaller surface area and the consequent weak van der Waals attraction. Similar to our previous work[5], here the reported side length, thickness and truncation distributions consider only the large plate population that assemble in our experimental conditions. Scale bars: 3 µm in (**a**), 1 µm in (**b**).

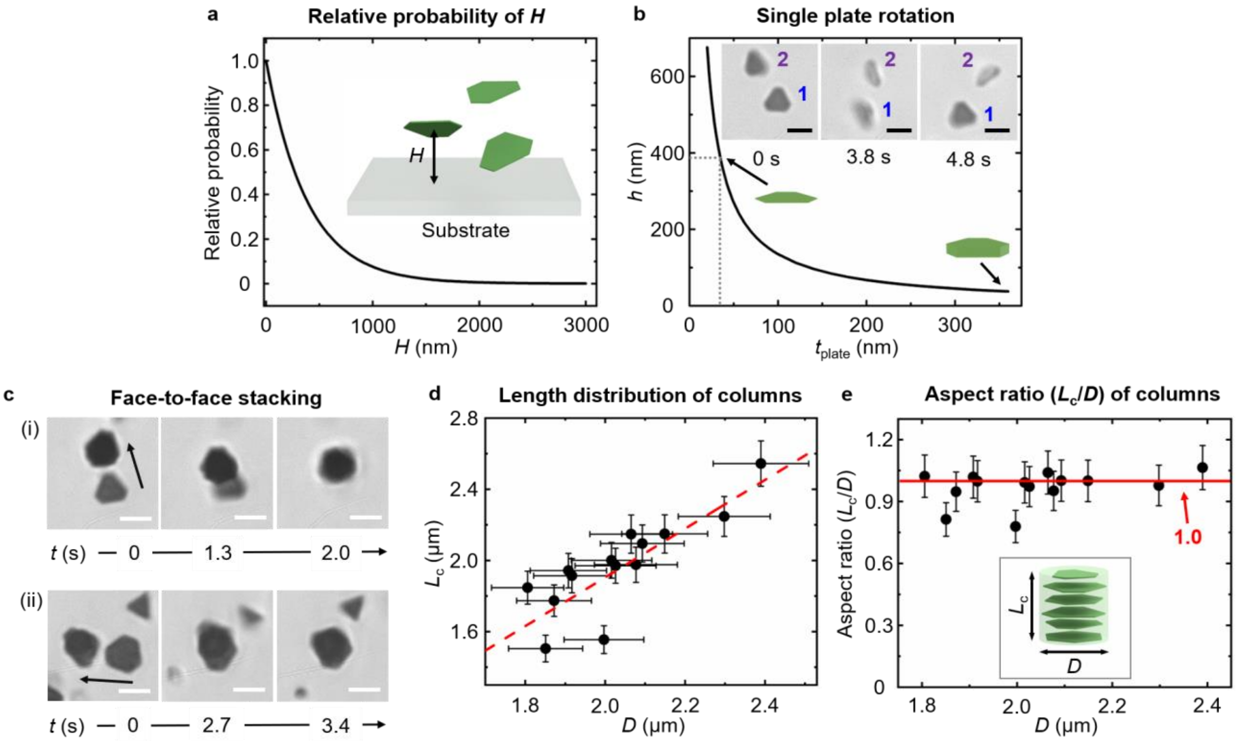

Supplementary Figure 2. The calculation of the fluctuation height of single plates and the optical microscopy characterization of the face-to-face plate stacking into columns. (**a**) A graph of calculated relative probabilities of plates residing at different heights (see Supplementary Note 1). The inset schematic shows the plate height H defined as the height of plate center-of-mass from the substrate. (**b**) A graph of calculated fluctuation height of plates as a function of the plate thickness (see also Supplementary Note 1). The inset schematics show the representative plates of different thickness (not drawn to scale). The thin plate on the left has the same dimension as that used in our hexagonal lattice assembly. The inset time-lapse optical microscopy images show the 3D rotation of two dispersed plates (labelled as “1” and “2”) in water. (**c**) Two sets of time-lapse optical microscopy images showing the plate face-to-face stacking process (indicated by the black arrows). (**d**) A graph of the length L_c distribution of assembled columns of different overall diameters D (see the inset schematic in (**e**)). Column lengths are measured from optical microscopy images when the columns are exhibiting out-of-plane rotations. The red dotted line is the linear fit to the data. (**e**) A graph of the aspect ratio distribution of assembled columns in (**d**). The column aspect ratio is defined as L_c/D. The horizontal red solid line is at an aspect ratio of 1.0 as the guide for the eye. The error bars in (**d**) and (**e**) are from the measurement errors in the optical microscopy images. Ionic strength in (**c**) to (**e**): 0.5 mM. Scale bars: 2 μm.

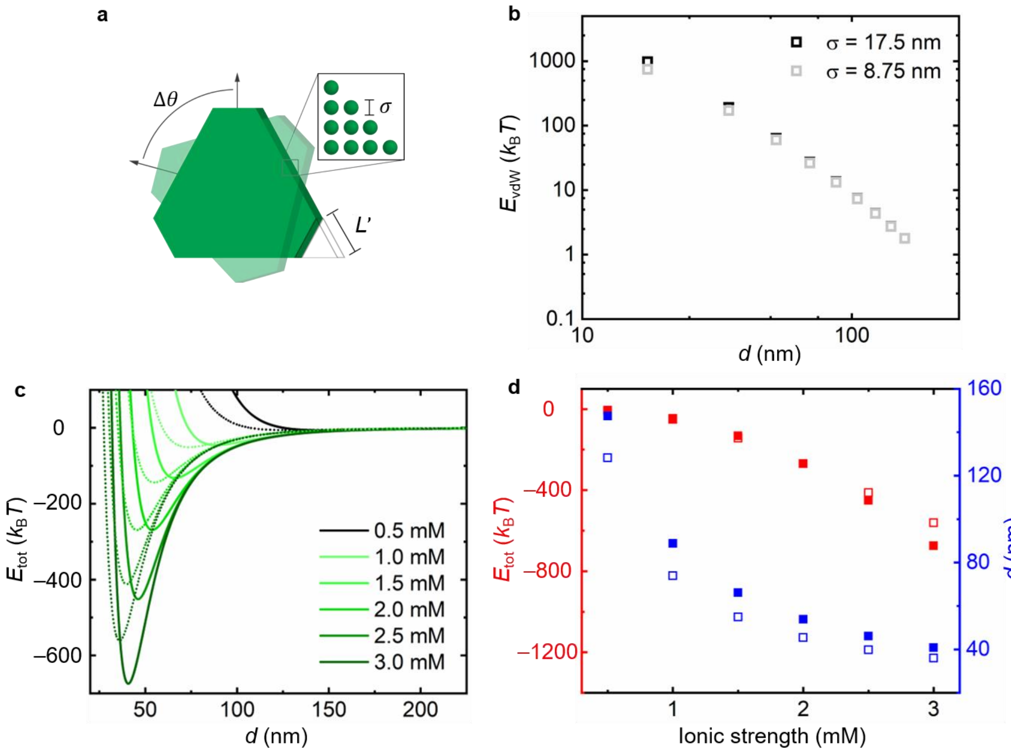

Supplementary Figure 3. Calculations of pairwise interaction and equilibrium interparticle d spacing of two face-to-face assembled silver plates using both analytical and discretized models. (**a**) A schematic showing the discretized model; $\Delta\theta$ is the relative rotation between plates, σ is the size of the discrete "bead," and L' is the extent of tip truncation. (**b**) The comparison of van der Waals interaction calculation using different bead sizes, which shows consistent results, especially in the experimental interaction range (~50–150 nm). (**c**) Net pairwise interaction energy of two stacked plates as a function of interparticle d spacing at different ionic strength conditions at $\Delta\theta$ = 0º. The solid lines are analytical model calculations and the dashed lines are discretized model calculations. (**d**) The interaction strength (red) and equilibrium interparticle distance d (blue) comparisons between analytical (solid squares) and discretized models (empty squares) at different ionic strength conditions at $\Delta\theta$ = 0º.

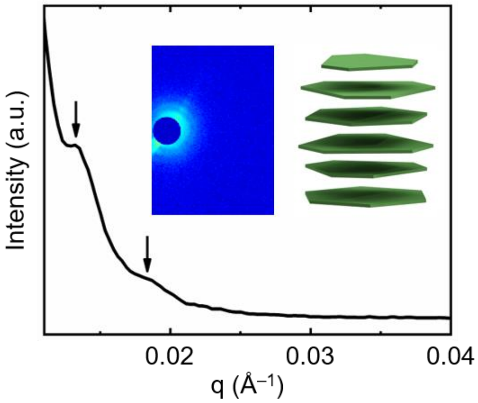

Supplementary Figure 4. Small-angle X-ray scattering (SAXS) spectrum of the column sample when the ionic strength is 0.9 mM. The black arrows indicate the peak positions. The left inset is the corresponding 2D scattering image from SAXS. The right inset is a schematic showing the 1D column structure assembled from plates. The peak positions are also listed in Supplementary Table 3.

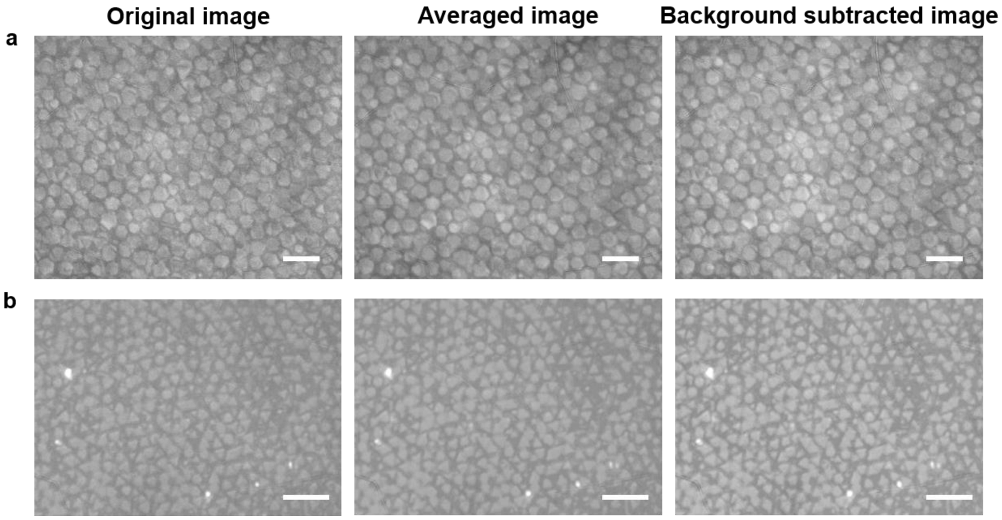

Supplementary Figure 5. The flowchart of image processing procedures. The image processing process illustrated by optical microscopy images including averaging and background subtraction used in Fig. 1e (**a**) and Fig. 4d (**b**). Scale bars: 5 μm.

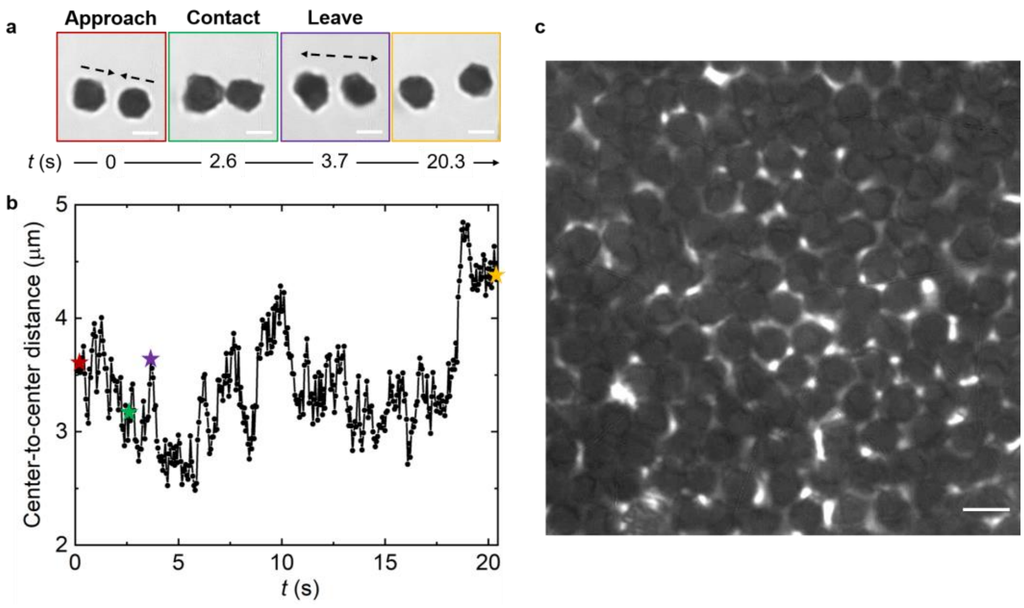

Supplementary Figure 6. Standing columns at low to high column concentrations. (**a**) Time-lapse optical microscopy images showing a representative column pair diffusing close and then apart due to the absence of strong column attractions at the same ionic strength as in the hexagonal lattice but at a lower column concentration. The black dotted arrows indicate the motion directions of this column pair. (**b**) A plot showing how the center-to-center distance between the columns in the pair (**a**) changes over time. The stars indicate the four time points color matched with the boxed images in (**a**). (**c**) An optical microscopy image showing the plates at intermediate column concentration. As shown in the image and Supplementary Movie 4, the empty space (bright regions) between each column helps visualize the stacking and rotation nature of plates in each column, further verifying the 3D structure in the assembly. Ionic strength: 0.5 mM. Scale bars: 2 μm in (**a**), 3 μm in (**c**).

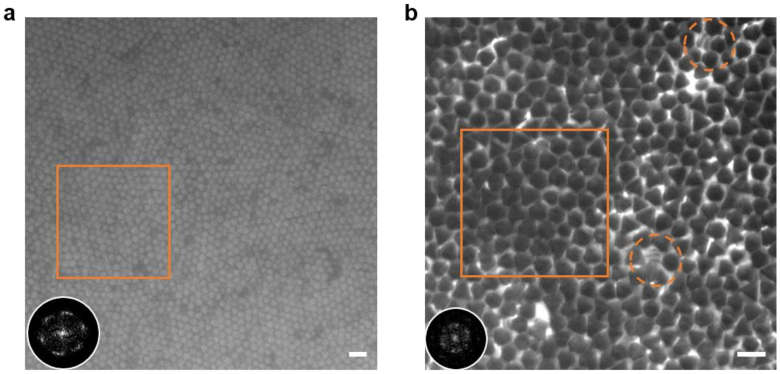

Supplementary Figure 7. Hexagonal lattices assembled from plates of different sizes. (**a**) An optical microscopy image showing the hexagonal lattice from relatively small plates (long side length L: 1.21 ± 0.15 μm, short side length L': 0.85 ± 0.17 μm; t_{plate}: 46 ± 10 nm, average truncation $\overline{m}$ = 0.71). The inset is the corresponding fast Fourier transform (FFT) of the image in the orange box. Ionic strength: 0.5 mM. (**b**) An optical microscopy image showing the hexagonal lattice from large plates at high plate concentration (long side length L: 2.18 ± 0.53 μm, short side length L': 0.93 ± 0.38 μm; t_{plate}: 30 ± 4 nm, average truncation $\overline{m}$ = 0.48). The inset is the corresponding FFT of the image in the orange box. The orange circles highlight the stacking nature of the columns when they are slightly tilted. Scale bars: 5 μm.

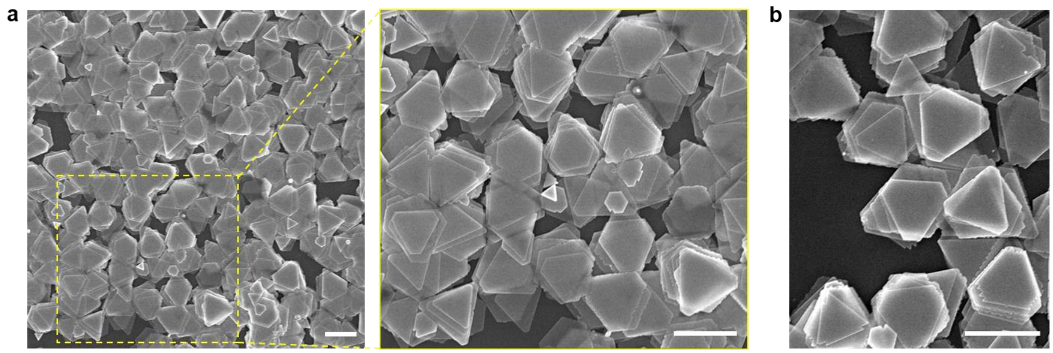

Supplementary Figure 8. SEM images (a) and (b) showing the 3D structures assembled from plates. The SEM images of the assembled 3D lattices after drying in air in the optical microscopy chamber, which show that plates stack face-to-face into columns, and these columns assemble laterally into 3D structures. The plate dimension is: long side length L: 1.35 ± 0.22 μm, short side length L': 0.64 ± 0.24 μm; t_{plate}: 28 ± 5 nm, average truncation $\bar{m}$ = 0.51. Scale bars: 2 μm.

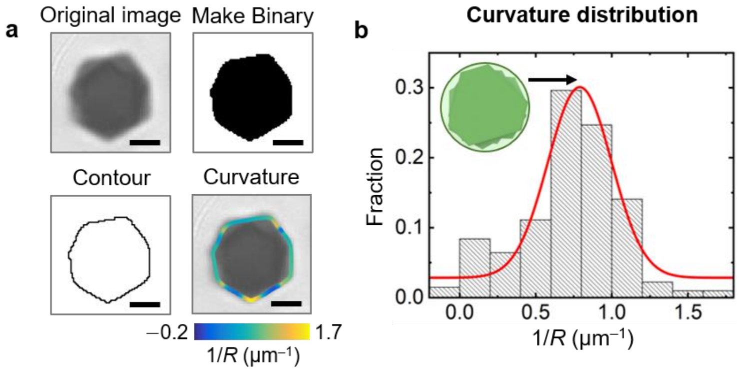

Supplementary Figure 9. Column curvature analysis procedures. (**a**) From the original optical microscopy image, we used the "Make Binary" function in ImageJ to binarize the image, the "Outline" function in ImageJ to recognize the contour, and the "Wand" function in ImageJ to get the XY coordinates of this contour, which serve as the inputs for the curvature calculation in MATLAB. The color bar represents curvature values. Scale bars: 1 μm. (**b**) A plot showing curvature distribution of all the column projections at 0 s, 0.2 s and 0.5 s. The red curve is the corresponding Gaussian fit, which gives a $1/R$ value of 0.79 ± 0.03 μm^{-1}. Thus, the radius of the column projection derived from the Gaussian fit is 1.27 ± 0.05 μm, close to the column projection radius measured directly from optical microscopy image (1.18 ± 0.10 μm, see Fig. 1b).

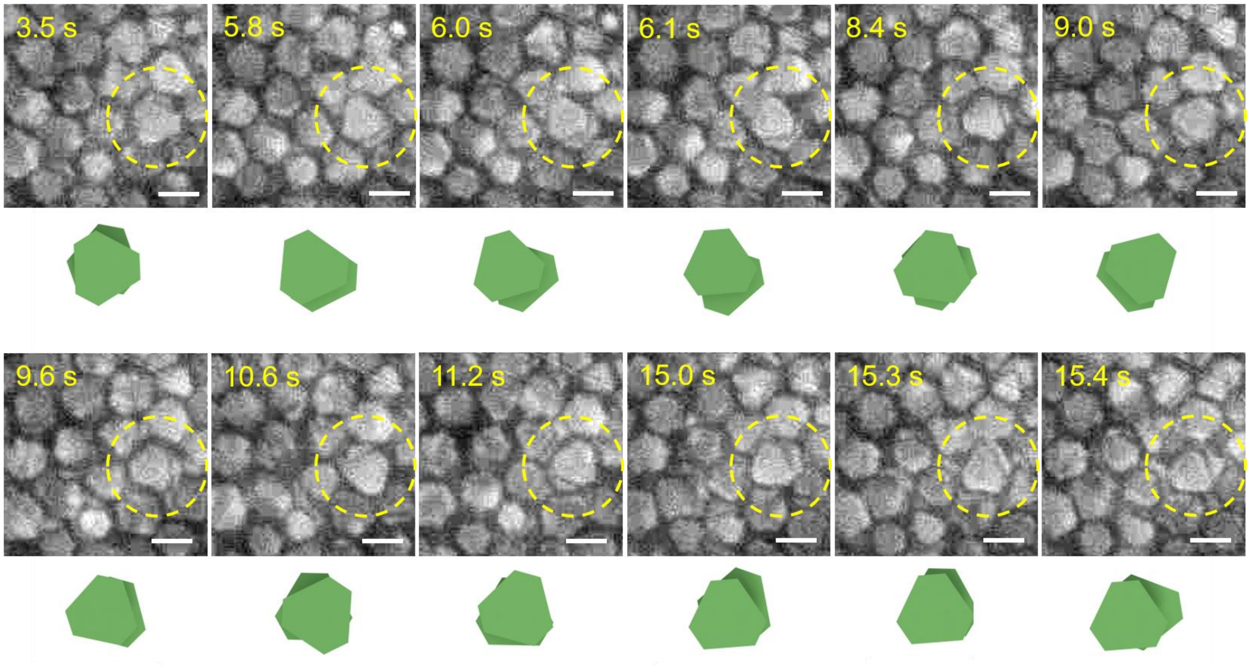

Supplementary Figure 10. Time-lapse optical microscopy images and corresponding schematics showing the visible first- and second-layer plates in the hexagonal lattice in Fig. 2c. Note that both top and bottom layers experience rotation over the time course shown. Scale bars: 2 μm.

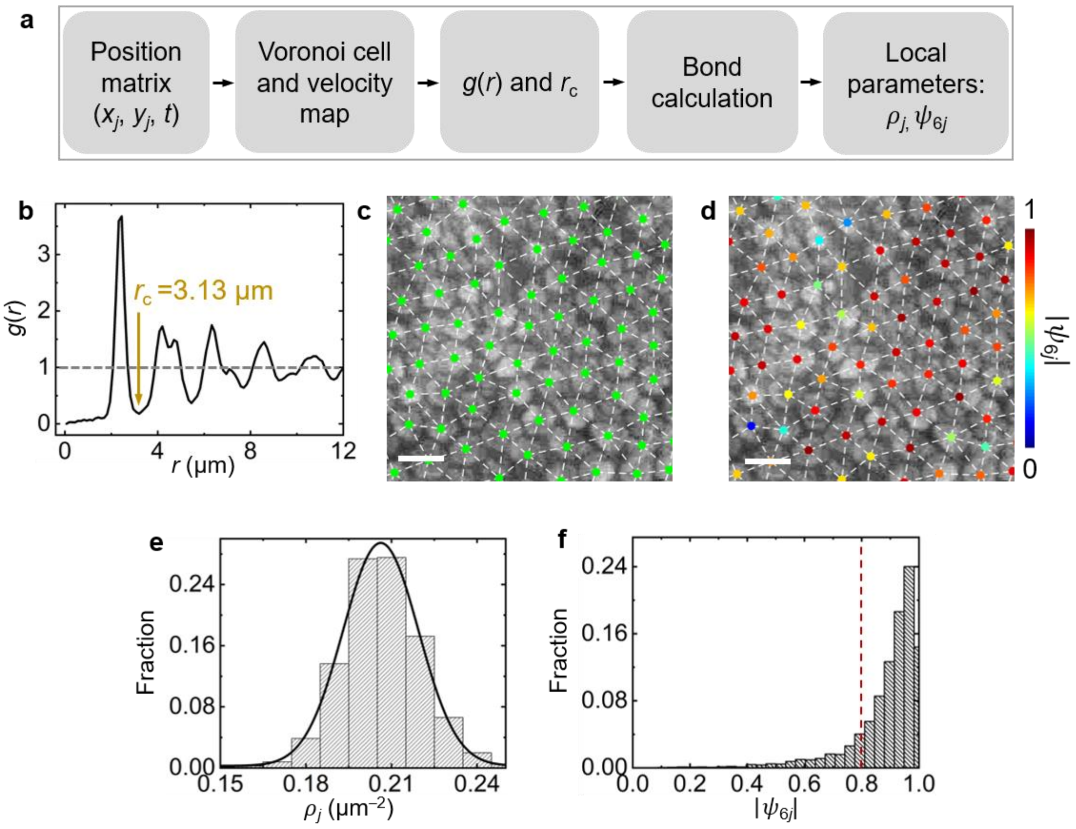

Supplementary Figure 11. The flowchart of the analysis procedure for the hexagonal lattice. (**a**) A workflow showing the whole analysis procedure. (**b**) Radial distribution function $g(r)$ calculated based on column positions by accumulating over 32 frames. A cutoff value r_c = 3.13 μm is measured from the first minimum after the first peak position (see the arrow). This cutoff value is used to determine if two columns are connected as nearest neighbors. (**c**) Bond network overlaid with the original optical microscopy image. (**d**) The optical microscopy image overlaid with bond network and the tracked center positions (dots color-coded according to the local order parameter $|\psi_{6j}|$ values). (**e**) Local density ρ_j distribution and (**f**) local structural order parameter $|\psi_{6j}|$ distribution over 32 frames. A Gaussian fit (black curve in (**e**)) to the local density distribution gives a peak position of 0.21 μm^{-2} (R^2 = 0.997), which is the same as the local density of a column in a hypothetical hexagonal lattice composed of closely packed circles circumscribing an average plate. Scale bars: 3 μm.

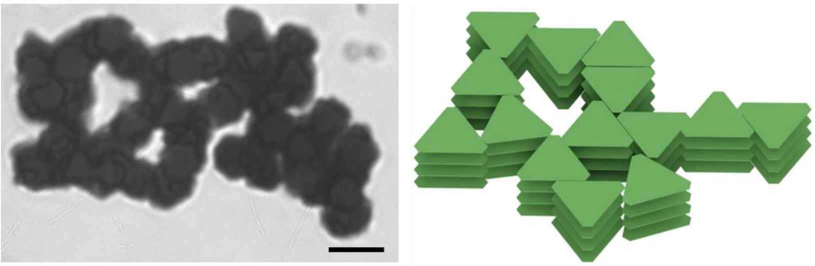

Supplementary Figure 12. A representative optical microscopy image showing irregular assemblies of columns at high ionic strength (2.0 mM). The plate sample here is the same sample (average truncation $\bar{m}$ of 0.55) used for the hexagonal lattice. Scale bar: 3 μm.

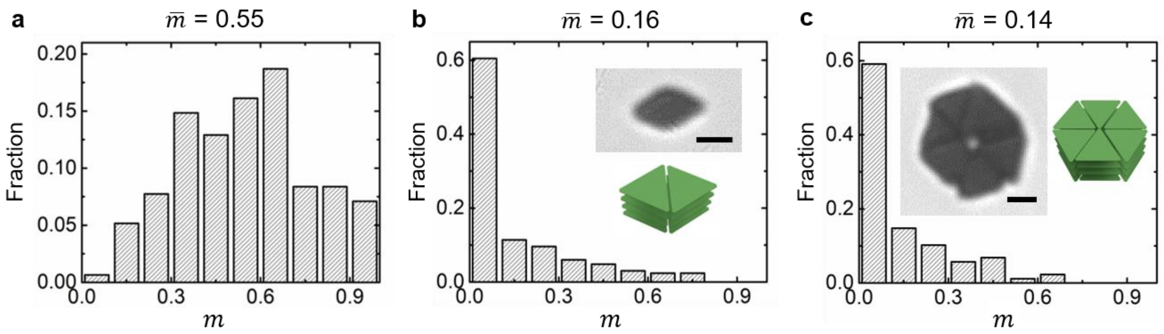

Supplementary Figure 13. The distributions of the extent of truncation (m) of the three types of plates whose SEM images were shown in Fig. 3a. (**a**) The plates of an average truncation $\bar{m}$ of 0.55, which are used for the hexagonal lattice assembly. (**b**) The plates of an average truncation $\bar{m}$ of 0.16. The inset is an optical microscopy image and the corresponding schematic of an assembled structure ("dimer") at 7 mM ionic strength. (**c**) The plates of an average truncation $\bar{m}$ of 0.14. The inset is an optical microscopy image and the corresponding schematic of an assembled structure ("hexamer") at 4 mM ionic strength. Scale bars: 1 μm.

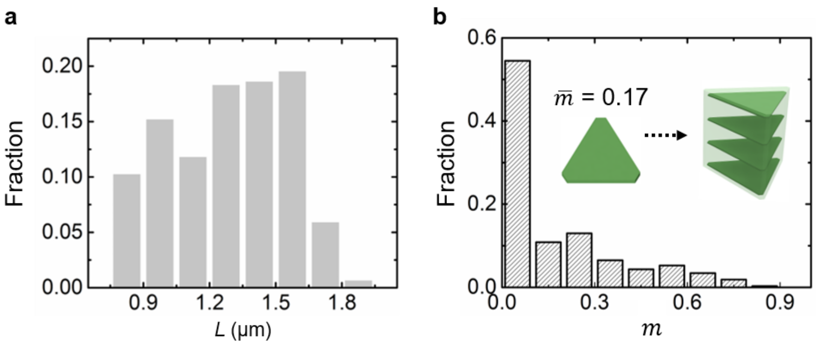

Supplementary Figure 14. Characterization of slightly-truncated silver plates used in Fig. 4 (long side length L: 1.28 ± 0.27 μm, average truncation $\bar{m}$ = 0.17) for the assembly of honeycomb domains. (**a**) The long side length distribution of plates measured from SEM images. (**b**) Truncation distribution of plates measured from SEM images. The inset schematics show a triangular column formation.

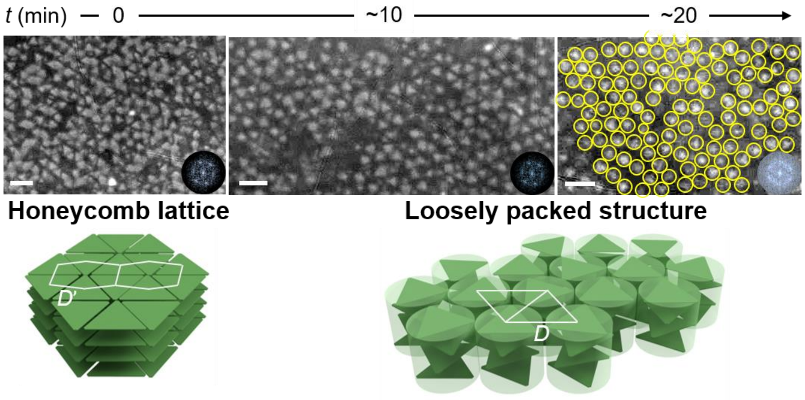

Supplementary Figure 15. The transition from honeycomb domains to a loosely packed structure upon an ionic strength decrease. Time-lapse optical microscopy images and schematics showing this transition from honeycomb domains to a loosely packed structure which exhibits hexagonal packings upon a decrease in ionic strength from 6.0 mM to 2.0 mM. The rightmost image was overlaid with yellow circles to highlight the column positions. The honeycomb domains at 6 mM are stable up to days. The insets in each image are the corresponding FFT of the images. The center-to-center distance between nearest column pairs measured from the optical microscopy images changes from 0.70 ± 0.09 μm (0 min) to 1.07 ± 0.11 μm (~20 min). The plates are the same as shown in the middle SEM image of Fig. 3a (long side length L: 0.92 ± 0.16 μm, short side length L': 0.12 ± 0.12 μm; t_{plate}: 22 ± 2 nm, average truncation $\overline{m}$ = 0.16). Scale bars: 2 μm.

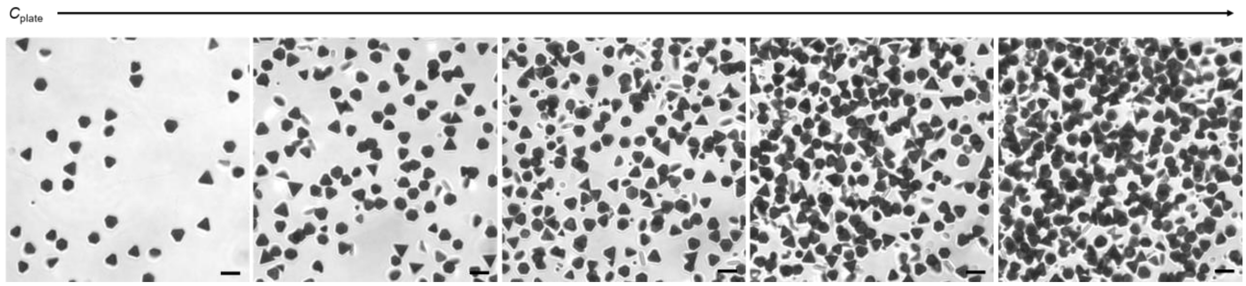

Supplementary Figure 16. 2D assembly of one layer of large plates. Optical microscopy images showing the 2D assembly at different plate concentrations in water, which did not show ordered structures. The plates have long side length L: 2.18 ± 0.53 μm, short side length L': 0.93 ± 0.38 μm; t_{plate}: 30 ± 4 nm, average truncation $\bar{m}$ = 0.48. Due to their large size, these plates mostly stay parallel with the substrate as one layer. Scale bars: 5 μm.

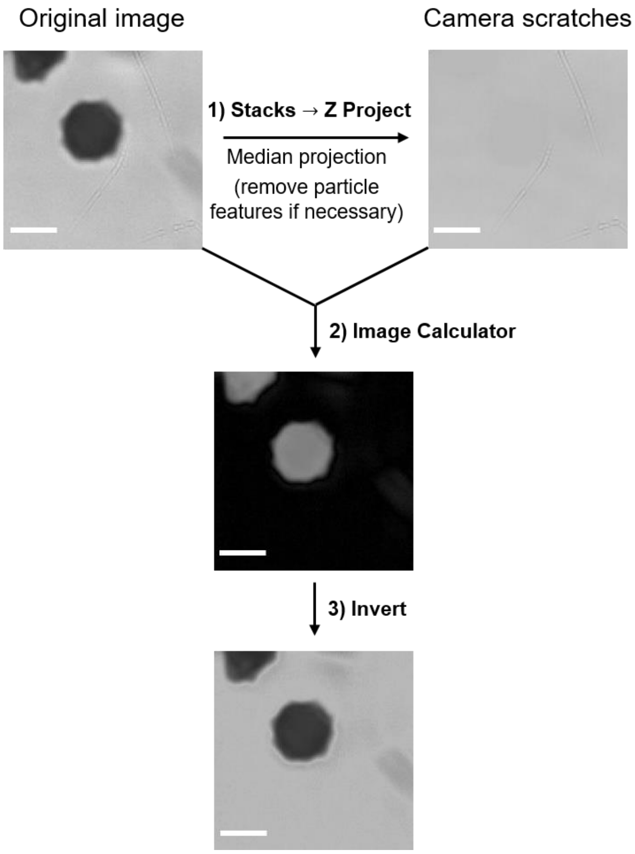

Supplementary Figure 17. The image processing procedures to remove defects. The procedures were conducted on Supplementary Movies 1 and 5 to remove the scratches on the camera, without changing any sample features in the movie. Scale bars: 2 μm.

Supplementary Tables

Supplementary Table 1. Synthesis conditions of silver plates of different side lengths and truncations.

The peak intensity in UV-Vis spectrum of the seed solution after dilution	Injection rate and injection time in each growth cycle	Shaking or stirring speed during growth (rpm)	The growth cycle to collect product	Long side length L (μm)	Average truncation $\overline{m}$
0.012	0.2 mL min^{-1}, 10 min	700 in cycle 1, 1000 in later cycles (shaking)	Cycle 5	1.49 ± 0.24	0.55
0.0024	0.4 mL min^{-1}, 5 min	700 in all cycles (shaking)	Cycle 5	1.21 ± 0.15	0.71
0.012	0.2 mL min^{-1}, 10 min	700 in cycle 1, 1000 in later cycles (shaking)	Cycle 7	1.35 ± 0.22	0.51
0.012	0.4 mL min^{-1}, 5 min	900 in cycle 1, 800 in later cycles (stirring)	Cycle 3	1.28 ± 0.27	0.17
0.012	0.4 mL min^{-1}, 5 min	1050 in cycle 1, 800 in cycle 2, 600 in later cycles (stirring)	Cycle 3	0.92 ± 0.16	0.16
			Cycle 4	1.54 ± 0.25	0.14
0.0024	0.4 mL min^{-1}, 5 min	700 in all cycles (stirring)	Cycle 4	2.18 ± 0.53	0.48

Supplementary Table 2. The parameters used in the plate–plate interaction calculation (Figs. 1d and 4e).

Interaction	Parameter	Value
van der Waals attraction*	Hamaker constant (H_0) at zero separation	4.0×10^{-19} J
	plate basal plane area (S)	3.27 μm^2
	plate thickness (t_{plate})	35 nm
electrostatic repulsion	carboxylate–thiol monolayer thickness (t_{ligand})	2.4 nm
	relative permittivity of water (ε)	78.5
	vacuum permittivity (ε_0)	8.854×10^{-12} F m^{-1}
	zeta potential of silver plates[5] (ζ)	-41.9 mV

*For the interaction calculations of the slightly truncated plates presented in Fig. 4e, the plate basal plane area and thickness values are 1.15 μm^2 and 22 nm. All the other parameters are the same as listed in Supplementary Table 2.

Supplementary Table 3. The calculated center-to-center d_c spacing value ($d_c = \frac{2\pi}{q_1}$), peak positions q_1 to q_4, and ratios between higher-order peak positions (q_2 to q_4) and the first-order peak position q_1 in SAXS spectra and the comparison of d_c obtained from SAXS, analytical and discretized models.

Ionic strength (mM)	d_c (nm)	q_1 (Å^{-1})	q_2 (Å^{-1})	$\frac{q_2}{q_1}$	q_3 (Å^{-1})	$\frac{q_3}{q_1}$	q_4 (Å^{-1})	$\frac{q_4}{q_1}$
0.9	142.8	0.0044*	0.0088*	2.0	0.0132	3.0	0.0184	4.2
0.9	130.9	From analytical model						
0.9	117.4	From discretized model						

*These q values were derived based on higher-order peak positions.

Supplementary References

1. Raşa, M. & Philipse, A.P. Evidence for a macroscopic electric field in the sedimentation profiles of charged colloids. *Nature* **429**, 857–860 (2004).
2. Sander, R. Compilation of Henry's law constants (version 4.0) for water as solvent. *Atmos. Chem. Phys.* **15** (2015).
3. Parsegian, V.A. *Van der Waals forces: a handbook for biologists, chemists, engineers, and physicists*, Cambridge University Press, (2005).
4. Hara, R., Fukuoka, T., Takahashi, R., Utsumi, Y. & Yamaguchi, A. Surface-enhanced Raman spectroscopy using a coffee-ring-type three-dimensional silver nanostructure. *RSC Adv.* **5**, 1378–1384 (2015).
5. Luo, B. et al. Polymerization-like co-assembly of silver nanoplates and patchy spheres. *ACS Nano* **11**, 7626–7633 (2017).
6. Kim, J., Ou, Z., Jones, M.R., Song, X. & Chen, Q. Imaging the polymerization of multivalent nanoparticles in solution. *Nat. Commun.* **8**, 761 (2017).
7. Liu, B. et al. Switching plastic crystals of colloidal rods with electric fields. *Nat. Commun.* **5**, 3092 (2014).
8. Frenkel, D. & Smit, B. *Understanding Molecular Simulation: from Algorithms to Applications*, Academic press, Vol. 1 (2001).
9. Zhang, T.H. & Liu, X.Y. How does a transient amorphous precursor template crystallization. *J. Am. Chem. Soc.* **129**, 13520–13526 (2007).
