# Supplementary material for: Hierarchical self-assembly of 3D lattices from polydisperse anisometric colloids
Source: Nat Commun. 2019 Apr 18;10:1815. doi: 10.1038/s41467-019-09787-6 (PMC6472373; doi:10.1038/s41467-019-09787-6)
Supplement: Supplementary file 3 — Description of Additional Supplementary Files [file 41467_2019_9787_MOESM3_ESM.pdf]

Description of Additional Supplementary Files

File Name: Supplementary Movie 1

Description: **Plates stack face-to-face into columns.** Two optical microscopy movies showing the plates stacking face-to-face into columns. The green arrows in each movie highlight the process of an incoming plate added to the “standing” columns. Image processing follows Supplementary Figure 17. Ionic strength: 0.5 mM. The movie is played at 17.2 frames per second (fps) in real time. Scale bars: 2 μm.

File Name: Supplementary Movie 2

Description: **Out-of-plane rotation of columns.** An optical microscopy movie showing the out-of-plane rotations of a column highlighted in the green circle. The column exhibits light and rectangular projections while lying on its side, and dark, polygonal projections while standing vertically on the substrate, verifying the stacking of plates in the column. Ionic strength: 0.5 mM. The movie is played at 17.2 fps in real time. Scale bar: 2 μm.

File Name: Supplementary Movie 3

Description: **A 3D hexagonal lattice assembled from polydisperse anisometric plates.** Left: an optical microscopy movie showing a three-dimensional hexagonal lattice. Right: the boxed region in the left movie overlaid with bond network (top, color-coded according to the local order parameter $|\psi_{6j}|$) and Voronoi cell representation (bottom, color-coded according to local density ρ_j). Ionic strength: 0.5 mM. The movie is played at 8.6 fps in real time. Scale bars: 5 μm.

File Name: Supplementary Movie 4

Description: **Assemblies at intermediate column concentration.** An optical microscopy movie showing the structures formed at intermediate column concentration, close to the hexagonal lattice formed at high column concentration as shown in Supplementary Movie 3. Ionic strength: 0.5 mM. The movie is played at 17.2 fps in real time. Scale bar: 3 μm.

File Name: Supplementary Movie 5

Description: **Columns of different effective shapes.** Top: An optical microscopy movie (left) showing the projection of a single column over time which can assemble into the hexagonal lattice, overlaid with its contour color-coded according to local curvature (middle) and a synchronized graph (right) showing the corresponding distribution of local curvature. Ionic strength: 0.5 mM. Bottom: An optical microscopy movie (left) showing the projection of a single column over time which can assemble into the honeycomb lattice, overlaid with its contour color-coded according to local curvature (middle) and a synchronized graph (right) showing the corresponding distribution of local curvature. Ionic strength: 3.2 mM. Image processing follows Supplementary Figure 17. The movie is played at 17.2 fps in real time. Scale bars: 1 μm.

File Name: Supplementary Movie 6

Description: **Plate rotations in a nearest column pair of the hexagonal and honeycomb lattices.** Top: An optical microscopy movie (left) showing two plates rotating in a nearest column pair (overlaid with red and blue arrows) inside the hexagonal lattice and a synchronized plot (right) showing the corresponding plate orientations (θ) as a function of time. Ionic strength: 0.5 mM. Bottom: An optical microscopy movie (left) showing two plates rotating in a nearest column pair (overlaid with red and blue arrows) inside the honeycomb lattice domain and a synchronized plot (right) showing the corresponding plate orientations (θ) as a function of time. Ionic strength: 3.5 mM. The top movie is played at 17.2 fps in real time and the bottom movie is played at 17.2 fps at a speed of 0.8 times real time. Scale bars: 1 μm.
